# Can tobacco use end in Europe?

**DOI:** 10.1093/eurpub/ckad068

**Published:** 2023-06-02

**Authors:** Pekka Puska

**Affiliations:** Finnish Institute for Health and Welfare (THL), Helsinki 34 F 00400, Finland

Tobacco is known to be globally the greatest single killer with some 8 million annual deaths. It is also great health risk to users and harms also nonusers and the society.^1^ Europe is the region with the highest level of tobacco use and consequently causing even higher share of deaths.^2^

For decades, health campaigns and policies have worked to reduce smoking. In view of the global background and the need for binding global action, the WHO Framework Convention on Tobacco Control (FCTC) was adopted in 2004 with most of the countries in the world as parties. The impact assessment of the FCTC showed significant gains in tobacco control and consequently some reduction in tobacco use has been observed.^3^

Western countries have in many ways led the way in tobacco control and the European Union has been active. Europe has a high prevalence of tobacco use, especially in Eastern Europe and among males, but the prevalence has clearly declined. However, the progress so far has been insufficient to reach the WHO target of 30% reduction in tobacco use in Europe by 2025.^2^

With the continuous amendments in tobacco policy and the declining trends already several years ago some tobacco control experts started to discuss what is the final goal—not just reduction. This led to the discussion on endgame, i.e. reaching the situation where tobacco use is so low (below 5%) that it is of little significance to public health.

Following this, many countries have adopted this target in their national tobacco strategy. Finland was the first country, and obviously still the only one, where this target is in the national legislation. In 2010, the Finnish Parliament decided in the first paragraph of the amended tobacco law that ‘the aim of this legislation is to end the use of tobacco products’.

The European health ministers in 2013 in the declaration of their Asghabat meeting asked for action towards a tobacco free European region. And recently the EU’s ‘Europe’s Beating Cancer Plan’ called for ‘Achieving a tobacco-free Europe’.^4^

Many meetings and papers have recently discussed the elements of the endgame.^5^ It is obvious that a prerequisite is an explicit government intention and plan. Another is ‘full implementation of the FCTC provisions’. But mere declarations are not enough.

Reduction of tobacco use is a social change process where legislation, health promotion and changes in tobacco use go hand in hand. Thus, dedicated and intensive continuation of the existing tobacco control work is needed. But also new innovations and activities targeting especially disadvantaged groups should be developed. At the same time, it is important to remember that price is the most effective instrument, and continuous and substantial increases are needed.^3^

The practical endgame work needs to involve different actors and the whole society. All public sectors should honour the FCTC Article 5.3. principle that ‘tobacco control policy should be protected from commercial and other vested interests’. The role and innovative work of NGO’s is particularly important to mobilize the population. This is because in the end of the day public opinion is the greatest push for effective legislation.

A relatively new question in the endgame is how to deal with the new nicotine products that maintain nicotine addiction, are harmful and interrelate with smoking. The international tobacco industry has especially in the Western countries shifted their marketing to these products.

The amendment of the Finnish tobacco law in 2016 included in the law also ‘end of use of nicotine products’. This is a big new challenge to expand national tobacco control and legislation, since marketing and increasing use of these products concern especially youth.

Tobacco endgame is a major global public health effort of this century, with success it will have a huge impact on global health and sustainable development.

*Conflicts of interest*: None declared.
